# An Intelligent Tool for Activity Data Collection

**DOI:** 10.3390/s110403988

**Published:** 2011-04-06

**Authors:** A. M. Jehad Sarkar

**Affiliations:** Department of Digital Information Engineering, Hankuk University of Foreign Studies, 89 Wangsan-ri, Mohyeon, Cheoin-gu, Yongin-si, Gyeonggi-do, 449-791, Korea; E-Mail: jehad@hufs.ac.kr; Tel.: +82-31-330-4627

**Keywords:** activity recognition, activity datasets, intelligent data collection, experience sampling tool, web mining, testbed

## Abstract

Activity recognition systems using simple and ubiquitous sensors require a large variety of real-world sensor data for not only evaluating their performance but also training the systems for better functioning. However, a tremendous amount of effort is required to setup an environment for collecting such data. For example, expertise and resources are needed to design and install the sensors, controllers, network components, and middleware just to perform basic data collections. It is therefore desirable to have a data collection method that is inexpensive, flexible, user-friendly, and capable of providing large and diverse activity datasets. In this paper, we propose an intelligent activity data collection tool which has the ability to provide such datasets inexpensively without physically deploying the testbeds. It can be used as an inexpensive and alternative technique to collect human activity data. The tool provides a set of web interfaces to create a web-based activity data collection environment. It also provides a web-based experience sampling tool to take the user’s activity input. The tool generates an activity log using its activity knowledge and the user-given inputs. The activity knowledge is mined from the web. We have performed two experiments to validate the tool’s performance in producing reliable datasets.

## 1. Introduction

Human Activity recognition has emerged as an active and challenging research area in ubiquitous computing during the last decade. A large number of activity recognition based applications have been investigated, e.g., patient monitoring system, employee monitoring system. A variety of activity recognition systems have been developed to meet the requirements of such applications [1–9].

When we talk about building an activity recognition system, we need to think about how to train the system for better functioning and how to evaluate the performance of the system. We need a large variety of real-world activity datasets such that we can use these datasets not only for training but also for evaluating the system’s performance.

However, collecting real-world activity datasets is not an easy task. We need to build testbeds to observe and accumulate the interaction information between users and their home environment. Testbeds are built by embedding a number of ambient sensors with a set of home appliances in the subject’s living environment and data is collected by observing the sequences of object usage (as the user interact with the object with embedded sensor) for activities.

There is a significant amount of effort needed in constructing a physical testbed to collect the real-world activity data [10]. Expertise and resources are needed to embed sensors with objects and to design controllers, network components, and middleware just to perform basic data collections. Another difficulty in building such testbed is in creating an effective way to annotate subject’s activities in an automatic and easy way. Therefore, acquiring real-world activity datasets by building a testbed is expensive and as a result, very few physical testbeds exist. In cases where real sensor data have been collected and analyzed, only rarely is this data made available to the research community.

Moreover, the range and variety of real-world datasets are limited to only one or two environments. The diversity of such datasets is extremely limited. In activity recognition research, it is important to validate the performance of the system with a diverse set of real-world activity examples. The algorithm tested with the datasets acquired from a variety of environment would be robust in general. It would therefore show superior performance (irrespective of the environment) in comparison with the algorithms validated with the datasets acquired from one environment.

Additionally, the datasets are relatively small, which has a variety of reasons: (1) it is difficult to find volunteers who would stay in a testbed and annotate their own activities; (2) even if we find such volunteers, they are required to stay in the testbed for long time to generate a reasonable amount of activity data. For example, in order to have 100 instances of an activity, volunteer(s) might need to stay in the testbed for 100 days; (3) the volunteers have to be focused all the time for accurate annotation of activities; (4) it could be very expensive to get a large dataset; (5) it would be difficult to formulate the appropriate object-usage interpretation.

Thus it is desirable to have an intelligent data collection method that is inexpensive, flexible, user-friendly, and also capable of providing large and diverse activity datasets. Before describing our contributions, it would be convenient to describe what an activity dataset is and what the entities of this dataset are. An activity dataset consists of a bunch of activity instances, each of which contains two types of information: the activity information and the corresponding object-usage information. The activity information is the activity name (or activity ID) and its duration (*i.e.*, start and end point). On the other hand, object-usage information is the set of object-usage (e.g., door, oven) and their corresponding start and end point.

Our motivation is to investigate how to comprise such a dataset without physically building the testbed. This would be the most inexpensive and may be the most feasible solution. Therefore, in [11] we propose a tool for collecting diverse activity datasets without actually building a testbed in real time. In order to generate an activity log (or instance), the tool first takes object-usage information from user and then produces an activity log using a predefined rule-based interpretation algorithm. It does not provide any mechanism for removal of the noises associated with the input. This paper however extends the tool by introducing an intelligent component for partially removing such noises. The component uses the web activity data for this purpose.

In short, the tool provides a set of web interfaces to create a web-based activity data collection environment. It also provides a web-based Experience Sampling method Tool (EST) by which a participant (or user) can supply his/her activity information by choosing the set of objects he/she interacted with and by providing their interaction sequence. The tool then transforms this information into an activity log using the activity knowledge it has already mined from the web. The contributions of this paper are summarized below:

1. We propose a web-based activity data collection tool which can be used as an inexpensive and fast alternative to collect diverse activity datasets without actually building a testbed in real time.
2. We propose a set of web interfaces to construct the activity data collection environment. We also propose a web-based EST to capture the participant’s object-usage information of an activity.
3. We propose an Intelligent Activity Generation Component (IAGC) which takes participant’s (or user’s) input and produces an activity log as output by partially removing the noises. For this intent it uses its existing activity data acquired from the web.
4. The IAGC mines the web activity data using a web mining algorithm proposed in [9].
5. We have performed two experiments to validate the tool’s performance in producing reliable datasets.

The rest of the paper is organized as follows. In Section 2, we review the previous works related to the real-world activity data collection tool. In Section 3, we give an overview of the proposed tool. This is followed by the detail descriptions of each of these components in Sections 4–6. In Section 7, we show the experiments we have performed to validate the tools performance in producing reliable datasets. In Section 8, we describe various issues associated with the tool. In Section 9, we conclude our paper with a direction of future work.

## 2. Related Work

There are a few tools that have been built for the real-world activity data collection. To the best of our knowledge, this work is the first of such tools that use web-based interfaces to construct the data collection environment and to collect activity information.

The activity data collection (using simple and ubiquitous sensors) procedure requires a series of actions: (1) select (or build) a home in which activity data will be collected, (2) select a set of home objects (*i.e.*, appliances) and embed sensors to these such that it is possible to determine the state of an object while interacted, (3) select the set of activities to observe and, (4) assign one or more participants to perform these activities in the testbed and annotate their own activity with the help of an EST.

To the best of our knowledge, Tapia *et al.* [5] first proposed the real-world activity data collection tools using simple and ubiquitous sensors. The authors deployed 77 and 84 sensors (equipped with reed switch) in two single-person’s apartments. The sensors were installed in everyday objects such as drawers, refrigerators, containers to record activation/deactivation events (opening/closing events) as the subject carried out everyday activities. The authors also provided an electronic EST in a portable computing device (*i.e.*, PDA) to the subjects. The EST prompts with a set of questions after every pre-specified period of time and the subject provides his/her answer by tapping one choice. The sensor data were collected by a base station and labeled using an EST.

Kasteren *et al.* [6] also proposed a real-world activity data collection tool that is similar to that of Tapia *et al.* [5]. The authors deployed 14 digital sensors in a house of a 26-year-old man, attached these sensors to doors, cupboards, a refrigerator, and a toilet flush. They proposed an innovative EST that use voice command to annotate an activity. They use a Bluetooth enabled handset combined with a speech recognition software (installed in a remote computer). During the data collection period, subject has to wear the headset and annotate their activity through voice command.

Chen *et al.* [12] also built a testbed in an apartment which consists of three bedrooms, one bathroom, a kitchen and a living/dining room. Their testbed is equipped with motion sensors placed on the ceiling. It also has temperature sensors along with custom-built analog sensors to provide temperature readings and usage of hot/cold water and stove burner. Additionally it has a power meter to record the total power usage and a set of switch sensors to monitor usage of the phone book, a cooking pot, and the medicine container. A student in good health acts as the participant. They stored sensor data (captured by a customized sensor network) in a SQL repository. They annotated the sensor data after the data collection procedure with the help of a Python Visualizer (PyViz). The PyViz can display events in real-time or in playback mode from the captured sensor event readings.

Wyatt *et al.* [13] deployed 100 RFID tags in a real home to make it a testbed. They have embedded these tags with a diverse set of objects like, faucets and remote controls. They had 9 participants with a wearable RFID reader. The participants were instructed to perform 14 activities. They were also instructed to write the order in which they have performed the activities in a diary. To establish the ground truth, the authors manually segmented and labeled (in offline) the activities with the help of these entries and perusal of the data stream.

Even though the aforementioned works have shown excellent promise in building the data collection tools, they had several limitations such as (1) special device was required to annotate an activity, and (2) they were unable to capture diverse set of data (since data collection was limited to one or two environments). Building a physical testbed of such is always expensive and therefore is the biggest drawback. Our tool on the other hand does not require any physical testbed which makes it applicable to almost all home environments. It is possible to obtain diverse set of data inexpensively from various environments. The proposed EST is web based and does not require any special device. Above all, the tool is extremely inexpensive and therefore can be used to as an alternative of real tesbed deployment.

## 3. Overview

**Figure 1.**
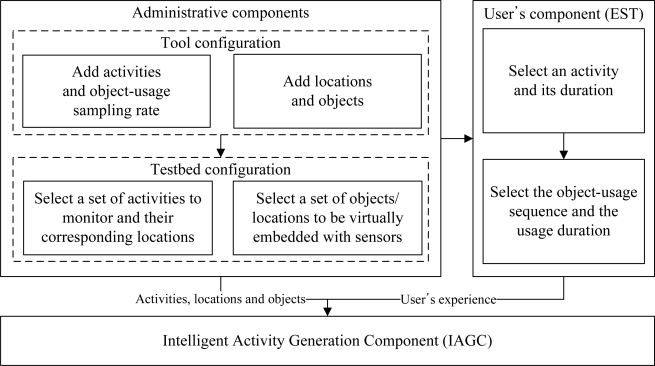
Overview of the tool for real-world activity data collection.

We have proposed an intelligent data collection tool that can be used to collect human activity data inexpensively. It uses a set of web interfaces to construct the data collection environment and to capture the user’s input of an activity. The tool combines the web activity data and the user’s input to generate an activity instance. It mines the web activity data using a web mining algorithm. It is inexpensive, flexible, and user-friendly by its very design. It has the ability to provide large and diverse activity datasets. The overview of the tool is shown in Figure 1. It consists of three major components:

1. Administrative component: The main objective of this component is to provide interfaces such that an administrator can configure the tool as well as the data collection environment.
2. User’s component: The purpose of this component is to provide interfaces such that a user can provide his/her experience (*i.e.*, interaction with the objects) after performing an activity.
3. Activity generation component: The objective of the IAGC is to transform a user’s experience to an activity log. It uses the web activity data for this transformation. The IAGC uses a web mining algorithm to acquire such data.

In the following sections we describe each of the components in more details.

## 4. Administrative Component

As described earlier, the objective of this component is to provide interfaces such that an administrator (or user with domain knowledge) can configure the tool and the testbed. He/she first configures the tool using the configuration interface by providing a set of activities, locations and objects it can deal with and the object-usage sampling rate (per activity). He/she then configures the testbed by choosing a set of activities to monitor, their corresponding locations and a set of objects per location. In this section, we describe each of these interfaces.

### 4.1. Tool Configuration

**Figure 2.**
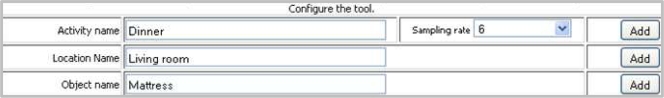
Tool configuration interface.

An administrator can configure the tool using an interface shown in Figure 2. He/she could add activities or locations or objects by simply giving their names in the respective fields and pressing the “Add” button. Additionally, he/she could provide the number of samples per activity such that a user can provide object-usage timing information per sample. We discuss more about this in Section 5.

### 4.2. Testbed Configuration

The tool offers two interfaces such that a user with little computer knowledge can configure the testbed for data collection. A user can configure the testbed by selecting a set of activities to monitor and by choosing a set of objects which could be used when giving the object-usage information regarding an activity.

**Figure 3.**
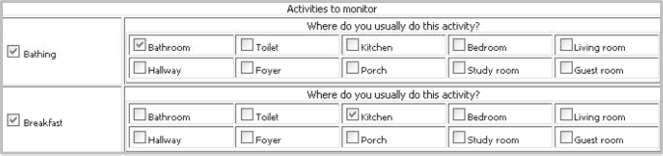
Testbed configuration: activity selection interface.

The interface to select a set of activities is shown in Figure 3. A user is expected to select an activity and the corresponding location(s) (*i.e.*, the room(s) to which an activity is usually performed). We take location as input because it provides important context information for activity recognition and could be very helpful to make the classification decision [14,15]. It is common to use a specific location to perform an activity. For example, the kitchen is for cooking and the bathroom is for bathing. The group of activities is limited for a given location. The set of activities and locations shown in the interface are retrieved from a table of a database saved while configuring the tool.

**Figure 4.**
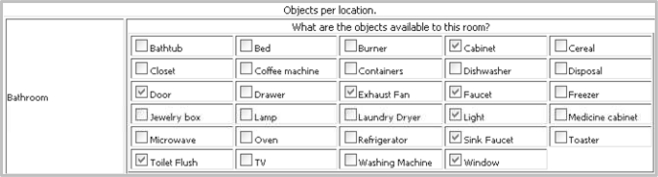
Testbed configuration: object selection interface.

The interface to choose a set of objects per locations is shown in Figure 4. The objects and locations shown in the interface are provided during tool configuration and testbed configuration respectively.

## 5. User’s Component

The purpose of this component is to provide an EST such that a participant can annotate his/her activity (along with its duration) and a set of object-usage sequence for that activity.

The tool uses a web-based inexpensive and user-friendly EST. We refrained from using any special device (which may not available in all environments) for EST since our goal is to make this a general purpose tool. Additionally, the web-based solution makes the data collection procedure ubiquitous. It means that a user is not required to be at home for inputting his/her activity information.

**Figure 5.**
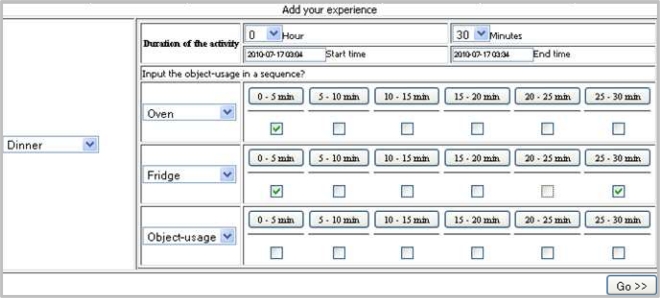
The experience sampling method tool.

A screen shot of the EST is shown in Figure 5. It consists of a number of selection menus, three of which are for providing the activity and its duration (*i.e.*, hours and minutes) and the rest are for providing object-usage sequence. It also consists of two text boxes (Start time and End time) which will be filled automatically upon providing the duration, however, user can also manually modify these. It also consists of a set of check boxes such that a user can provide the object-usage scenario.

The set of objects are the objects available to the location(s) in which the activity is performed. Therefore, the set of objects can be automatically changed when the selected activity is changed. The user must first select the activity from the activity selection menu. Upon selection of the activity, one row of object-usage becomes visible. The next row will only be visible if user chooses an object from the object-usage selection menu and the next will be shown if he/she selects another object and so on. The maximum number of object-usage would be the total number of objects available to the location in which the activity is performed. In this way we make the redundant substances invisible from user.

As shown in the screen shot, we divide the total activity period (say, *T*) for object-usage into a fixed number of slots, say *n*. A user needs to provide an object-usage duration through the interface. To facilitate the user input, the interface form contains a check box for each time slot. The user will check the box corresponding to a time slot if the object under consideration is used any time within the slot. For example, consider *n* = 6 and *T* = 30 min. Then each slot will consists of $\left(\frac{T}{n}=\frac{30}{6}=\right)\;5\;\text{min}$ (*i.e.*, 0–5 min, 5–10 min, 10–15 min, 15–20 min, 20–25 min, and 25–30 min). An interface form for the above setting is shown in Figure 5, where user has selected using both Oven and Fridge within 0–5 min slot and only Fridge in 25–30 min slot.

Generally, the interpretation of the object-usage input for a checked time slot is that, the object was in use (for the whole duration or a fraction of that) within the time slot. For example, for the time slot 0–5 min (in Figure 5) the Oven usage is found checked which interprets that for further time granularity of this slot (*i.e.*, 1st min, 2nd min, 3rd min, *etc.*) the object was constantly used.

After providing all the information, a user could add his/her experience. The EST stores all the given object-usage and their corresponding intervals with some random noises into a repository as raw data. The random noises make the data as close as possible to the real-world. In real-world activity scenario, it is possible to use one or more objects inadvertently that are not related to that activity (e.g., living room’s light is on while cleaning kitchen). Additionally, the EST sends these raw data to IAGC for generating the actual activity sample.

Although it is possible to add experiences anytime (e.g., at the end of each day), we expect a user to provide his/her experience at the end of each activity. The EST would beep in every 10 min to remind the user to provide his/her activity if it is finished. Providing activity information at the end of an activity would ensure a correct scenario of object-usage sequence for that activity. It also reduces user’s effort in providing their experience.

## 6. Intelligent Activity Generation Tool (IAGC)

A participant will not be able to provide precise object-usage duration with the proposed EST described above. It is possible to change the EST in a way such that a participant could provide close to precise object-usage duration. However, in that case he/she has to recall the exact duration of usage for all the objects he/she used. This will be extremely inconvenient for the participant since the number of object-usage could be overwhelming. This in turn will make the activity information (or experience) error-prone. Therefore, instead of depending on the participant to provide the reliable object-usage timings, we use an intelligent component (we named it IAGC) to generate such. The component takes the partial timing (or noisy timing) as input from the participant and produces a reliable timing.

**Figure 6.**
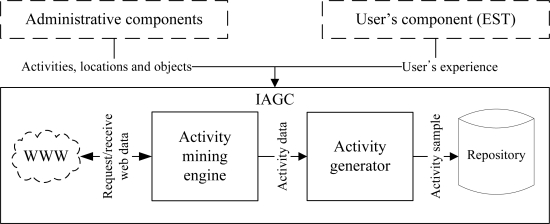
Intelligent Activity Generation Tool.

The overview of the IAGC is shown in Figure 6. It consists of two main modules, activity generator and activity mining engine. The activity generator transform the participant’s input into an activity sample using the mined data from the web. The IAGC uses the activity mining engine to mine activity data from the web. In the following subsections we described each of these modules in details.

### 6.1. Activity Generator

It is possible to interpret a checked slot as the constant use of an object (*i.e.*, for all the time units in the slot). However, the real-world scenario may be different from the above scenario in the sense that the object might not actually be used continuously for the whole period of a time slot. It is also possible that the usage of the objects might overlap partially or exclusively. For example, it is quite likely that the usage of a refrigerator and an oven might overlap partially and the usage of light and stove will overlap completely. The constant usage interpretation would wrongly interpret such situation. Therefore, we have introduced an intelligent activity generator module to deal with such circumstances intelligently. In short, it uses a scheme of reflecting the object-usage probabilities on interpreting the object-usage timings.

Let *d*_1_, *d*_2_, . . ., *d_n_* represent the slots where each slot *d_i_* is the combination of *t*_1_, *t*_2_, . . ., *t_m_*, where *t_i_* is a time unit and each slot consists of *m* time units. Considering these parameter settings and based on the above discussion we present our proposed object-usage interpretation scheme in Algorithm 1. The algorithm takes object-usage information such as set of objects, set of slots (*d*_1_, *d*_2_, . . ., *d_n_*) and number of unit time per slot (*t*_1_, *t*_2_, . . ., *t_m_*) as input. It outputs the object-usage states (in terms of unit time) for the given activity time period.

The activity generator first computes the probabilities of the objects used for the given activity, *a*, using the following formula,

(1) $$P\left(o_{\mathrm{ij}}|a\right)=\frac{\mathrm{freq}\left(o_{\mathrm{ij}}|a\right)}{\sum_{k}\mathrm{freq}\left(o_{\mathrm{ik}}|a\right)}$$

where, *P* (*o_ij_*|*a*), is the probability of an object, *o_j_*, if it is used in the duration, *d_i_*, *freq*(*o_ij_*|*a*) is the object-usage frequency for a given activity which is mined from the web by the activity mining engine (more about this are discussed in the following subsection). The probability, *P* (*o_ij_*|*a*), is 1 if *o_j_* is the only object used in the duration *d_i_*.

**Algorithm 1:**
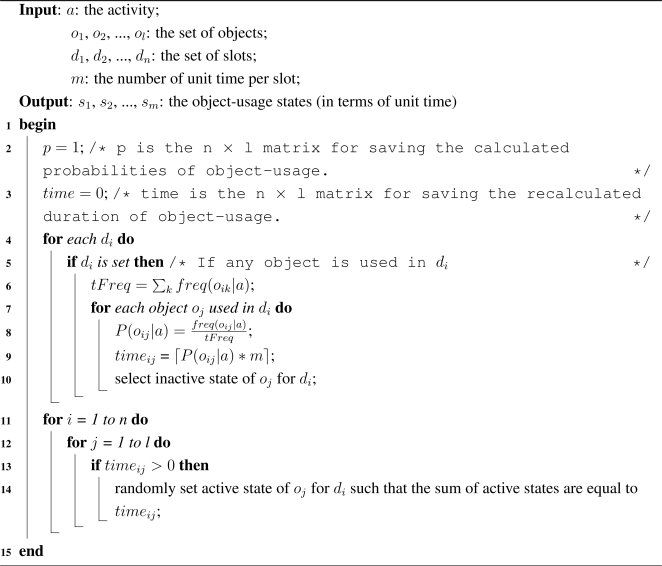
Object-usage interpretation algorithm.

The activity generators then redefine the actual time, *time_ij_* of an object-usage, as,

(2) $${\mathrm{time}}_{\mathrm{ij}}=\left\lceil P\left(o_{\mathrm{ij}}|a\right)*m\right\rceil$$

Finally, the activity generator uses the *time* matrix to set the active states of an object within a duration, *d_i_*, while interpreting the states of the objects. It gives more active states to the objects, whose probabilities are higher in a parallel environment (two or more objects are used within a same duration). For example, if an oven and a refrigerator are used in the same time slot, the activity generator provides more active states for the oven if the probability of using oven is higher than of refrigerator for cooking. The notion behind this method is that in real life, it is more likely that an object with high usage probability will be used more frequently than the one with low usage probability.

It is to be noted here that prior to taking activity data from user, the values for *n* and *T* are chosen by the administrator. The labels of the buttons in the interface form (e.g., 0–5 min, 5–10 min, *etc.*) are automatically adjusted according to the values of these two parameters. It is quite natural that we can increase the input flexibility in choosing lower value for *n*. However, such lower value of *n* may interfere the projection of real object-usage scenario. Therefore, the administrator is advised to choose a value for *n* by considering a trade-off between the two performance factors.

### 6.2. Activity Mining Engine

The advancement of the Internet and the WWW encourages millions of individuals to compose billions of web pages with varieties of contents [9,16]. A considerable number of pages describe in detail how to perform daily life activities. Such pages not only describe the activity but also depict where to perform and what objects to be used [9].

It is possible to use these pages to train an activity recognition system [9,13,17]. In [9], we have shown that how to mine activity data from such pages and use these as the basis of training data. In this paper we adopt the mining technique to determine the relationship between an activity and a set of objects.

As shown in Equation (1), the probability of an object-usage given an activity (*i.e.*, *P* (*o_ij_*|*a*)) is calculated by counting the relative frequencies. The goal of the mining engine is to provide these relative frequencies.

**Algorithm 2:**
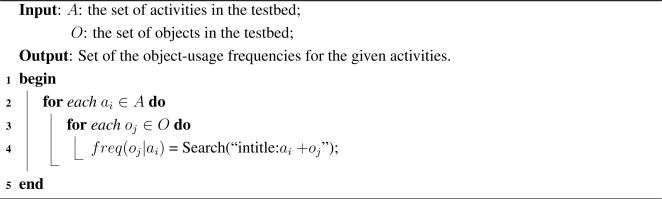
Activity mining engine.

For this purpose the activity mining engine uses a straightforward algorithm as shown in Algorithm 2. Given a set of activities and objects, the algorithm mines the web activity data using a search engine. It uses the page count returned by the search engine for a given query as the frequency. For example, the frequency of using Oven given Cooking, *i.e.*, *freq*(*Oven*|*Cooking*), is the page count returned by the search engine if we search with the following query, “intitle:*Cooking* +*oven*”.

**Figure 7.**
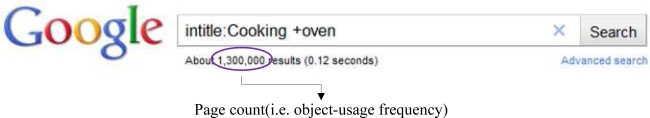
Object-usage frequency mining example.

An example is shown in Figure 7. Using query like, “intitle:*a_i_* +*o_j_*”, we are instructing the search engine to search only the pages which have the activity name (e.g., Cooking) in their title and the object name (e.g., Oven) in their body text.

### 6.3. Activity Generation Example

In this section we describe the process of generating an activity log with the help of the following example: a participant cleans a room with a vacuum cleaner for 20 min. He gets the vacuum cleaner from the closet, cleans the room and put it back into the closet.

**Figure 8.**
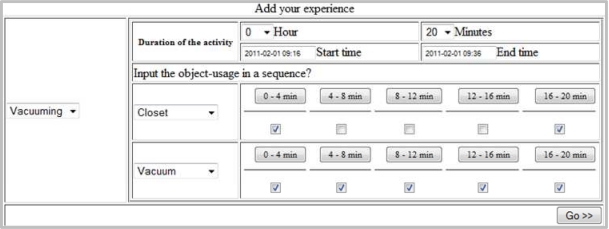
Inputting activity information using EST.

As we can see in this example, two objects are being used, closet and vacuum cleaner. The participant would like to input this activity information with the EST. Let us consider that the EST provides 5 timing slots, *d*_1_, *d*_2_, *d*_3_, *d*_4_ and *d*_5_ for vacuuming (*i.e.*, administrator has chosen *n* = 5 for this activity during the tool configuration). The participant will check all the slots for vacuum cleaner. He/she will check the 1st and 5th slot for both closet and vacuum. The scenario is shown in Figure 8.

If data related to vacuuming are provided to the EST then it stores and sends them to IAGC including one or more random noises (described in Section 5). Let the random noise in this case be the burner which is set active on for the 1st and 2nd slot. The EST representations of the object-usage are shown in Table 1.

After getting inputs from EST, the IAGC transforms them into an actual activity log with the help of the mined data. Let each slot, *d_i_*, consist of 4 units of time, *t*_1_, *t*_2_, *t*_3_ and *t*_4_. Let the mined object-usage frequencies of closet, vacuum and burner for vacuuming be 1,130, 82,600 and 267 respectively. If all three objects are used in parallel, then the probabilities of object-usages will be 0.014, 0.983 and 0.003 respectively. A probable output of IAGC is shown in Table 2 (each shaded cell represents an active state). As shown in Table 2, IAGC sets *d*_1_*t*_1_ and *d*_5_*t*_4_ as the active states for closet, because it can have at most 1 active state per slot (applying Equation (2)). Similarly, it sets *d*_1_*t*_1_ and *d*_2_*t*_2_ as active states for burner. However, it sets *d*_1_*t*_1_, *d*_2_*t*_2_, ... *d*_5_*t*_4_ as the active states for vacuum, because the total number of active states per slots for this object is 4.

## 7. Evaluation

We have performed two experiments to validate the performance of the tool. The objective of the first experiment is to train and evaluate a system’s performance with the help of an activity dataset acquired by the proposed tool. The objective of the second experiment is to see the performance of the activity generator for producing a dataset by taking input from an existing activity dataset (acquired from a real testbed). This section is organized as follows: we first describe the setup we have used for the web activity data mining and then describe the experiments.

### 7.1. Setup for the Web Activity Data Mining

As described earlier, the tool uses a search engine to mine the web activity data. In this experiment we used Google since this is the most popular web search engine worldwide [18,19]. Google is designed to retrieve relevant document from the World Wide Web (WWW) based on user’s query. We used the site, http://ajax.googleapis.com (developed by Google for applications to retrieve data from the Google server asynchronously), instead of the original site, http://www.google.com/, to mine the activity data. For example, to mine the frequency of “Oven” given “Cooking” (*i.e.*, *freq*(*Oven*|*Cooking*)), the tool would send a query as, http://ajax.googleapis.com/ajax/services/search/web?v=1.0&q=intitle:Cooking+Oven. In response, Google would return a page that would contain the formatted results like, the estimatedResultCount, the links of few (usually 4) result pages, the link for more results *etc*. The tool uses the field estimatedResultCount as the frequency. Searching with Ajax would retrieve a bit old data with respect to the original site. It is to be noted here that Google would not allow automated search using their original site.

### 7.2. Experiment I

#### Testbed Setup

As we discussed before, the goal is to make the datasets as inexpensive as possible. Therefore, we did not hire any participants, instead we select few volunteers. We have managed to convince one volunteer so far. The volunteer does not have any relation with our research group. She is a 28-year-old girl who lives in an apartment with her parents and two kids. We had a meeting with her for about two hours. During this time we educated her about our data collection tool, setup her individual environments and told her to play with the EST in front of us. It was easy to teach her since she was familiar with the web forms. She managed to setup her environment by herself.

Thirty objects in five different locations were chosen to acquire object-usage information from the user. Although her apartment consists of three bedrooms, three bathrooms, two balconies, one hallway, one kitchen, one living and one dining, we only consider one bedroom, one bathroom, one hallway, one kitchen, one living and one dining room. Each of the objects was given unique identification numbers. The list of the objects along with their locations are given in Table 3.

The testbed was set to record eight activities: breakfast, lunch, dinner, cooking, toileting, bathing, watching TV, sleeping and going out.

#### The Acquired Dataset

During the time of conducting the experiments, 10 days’ data were annotated. The dataset consists of two tables: activity table and object-usage table. Each of the entries of the activity table consists of the activity start-time, end-time and ID, and each of the entries of the object-usage table consists of the object-usage start-time, end-time and ID. An example of these tables are shown in Table 4.

After acquiring the dataset and carefully reviewing it, we have found a few problems. For example, the participant made mistakes while inputting start and end time of an activity or an object-usage, and some of the activities has no object-usage information. We have removed these faulty entries while preparing the dataset for experiment.

#### The Algorithm We Used

To validate the applicability of the dataset we use an activity recognition algorithm we proposed in [20]. The algorithm uses a Naive Bayesian based activity classifier and works in two layers. In first layer it uses location-and-object-usage based model classify a group of activities and in second layer it uses object-usage based model to classify an activity from that group. Interested readers are referred to [20] for more information. The shorter version of the algorithm will also be found in [9].

#### Evaluation Criteria

In this experiment the object-usage are divided in window size of length Δ = 1 min. This window size is long enough for discriminating and short enough for providing high accuracy labeling results [6]. We train the algorithm using both the real-world and the web activity data, and tested it with the real-world activity data. We use the web mining approaches proposed in [20] to collect the web activity data. Interested readers are also referred to [9,20] for more information.

Two types of accuracy measurements are used for evaluation: Timeslice accuracy and Class accuracy [6]. The Timeslice accuracy is measured by,

$$\frac{\sum_{i=1}^{N}{\mathrm{detected}}_{i}==\mathrm{true}}{N}$$

and Class Accuracy is measured by,

$$\frac{1}{C}\sum_{c=1}^{C}\left\{\frac{\sum_{i=1}^{N_{c}}{\mathrm{detected}}_{i}==\mathrm{true}}{N_{c}}\right\}$$

where, *N* is the total number of activity instances, *C* is the number of classes and *N_c_* is the total number of instances for class *c*.

#### Results

**Figure 9.**
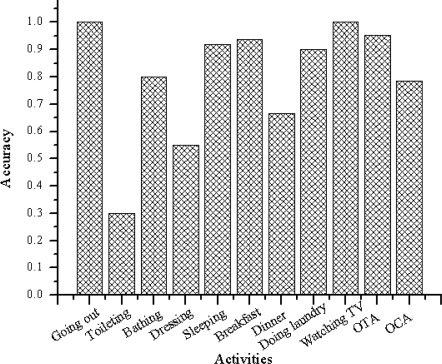
Algorithm’s (trained using the web data) accuracies per activity. The rightmost two bars compare the overall timeslice accuracy (OTA) and the overall class accuracy (OCA).

**Figure 10.**
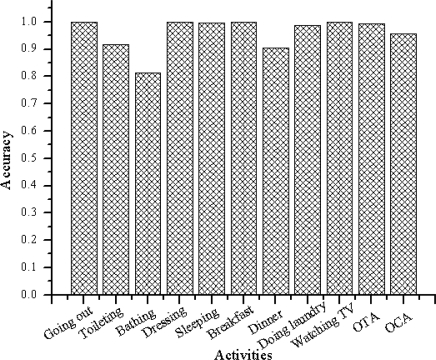
Algorithm’s (trained using the real-world data) accuracies per activity. The rightmost two bars compare the overall timeslice accuracy (OTA) and the overall class accuracy (OCA).

Figures 9 and 10 summarize the accuracies per class. Figure 9 shows the output when the algorithm is trained using the web activity data. Figure 10 shows the output when the algorithm is trained using the real-world activity data. The rightmost two clusters compare the overall timeslice accuracy (OTA) and the overall class accuracy (OCA).

As shown in Figure 9, the activity recognition system achieves overall class accuracy of 78.62% and overall time slice accuracy of 95.23% when learned using the web activity data. It achieves overall class accuracy of 95.73% and overall time slice accuracy of 99.27% when learned using the real-world activity data.

Table 5 shows the confusion matrix generated by the classifier when it is learned with the web activity data. It is observed that the classifier makes more confusion between the activities performed in a same location using similar objects. This is expected because the objects within that location are equally likely to be used for these activities. For example, as we can see in Table 5, the classifier made more confusion between “Toileting” and “Bathing” because, these two activities were performed in a same location (*i.e.*, “Bathroom”), and the number of distinguishing objects are low.

It is also observed that the OTA and OCA show different accuracy result. The reason are the number activity instances and their durations are imbalanced between activity classes. For example the number of occurrences (provided by the participant) of “Sleeping” are 10 and its duration is 7–10 h. On the other hand the number of occurrences of “Toileting” is 34 and its duration is 1–10 min. As described earlier, in this experiment the object-usage are divided in window size of length Δ = 1 min. Therefore, the total instances (the activity recognition system has sliced) of “Sleeping” are more than 4,200 and the total instances of “Toileting” are less than 340. As the activity recognition accuracy for “Sleeping” is higher than that of “Toileting”, the OTA and OCA are influenced to show different result.

### 7.3. Experiment II

The objective of this experiment is to see the performance of activity generator in producing a dataset by taking input from an existing activity dataset (acquired from a real testbed). In other words, the activity generator takes activity name, duration and object-usage scenario as input from an existing dataset and by manipulating this information produces a new dataset of its own. We compare these two datasets to validate the performance of the activity generator.

The dataset we use are acquired by Kasteren *et al.* [6] at Intelligent Systems Lab Amsterdam (ISLA) (we call it ISLA dataset). They have deployed 14 digital sensors in a house of a 26-year-old man, attached these sensors to doors, cupboards, a refrigerator, and a toilet flush. There are seven activities (chosen from Katz ADL index [21]) in the dataset. They have collected data for 28 days.

A different *n* (*i.e.*, number of slots, described in Section 6) is chosen for each of these activities as shown in Table 6. Each of these slots consists of *m* time units of 1 minute each. The *n* is chosen depending on the duration and the number of object used for an activity. For example, *n* = 2 is chosen for “Drink” since its duration and number of object is very low. Similarly, *n* = 2 is chosen for “Sleeping” since the number of object-usage is very low.

The activity generator produces the dataset as follows. For each activity, *a_i_*, in the existing dataset, the object-usage interpretation algorithm takes following input: *n* and *m* and the set of object-usage, *o*_1_, *o*_2_, . . ., *o_l_*, per slot. It then produces the object-usage states for that activity.

After that we compare the object-usage scenario of these two datasets to see how accurate the IAGC is in producing the dataset. The results are shown in Table 6. The activity generator achieves overall accuracy of 83%.

It is observed that the object-usage scenario of an activity is more accurately generated by the activity generator if the most appropriate or most frequently used objects (generally used for that activity) are used while performing that activity. For example, the activity generator achieves overall accuracy of 96% for “Drink” since two important objects, “Cup” and “Fridge” of this activity are frequently used. On the other hand, for “Sleeping” the activity generator achieves low accuracy (*i.e.*, 61%) because no important object (e.g., mattress) of this activity was tagged with sensor. The activity generator depends on the probability of an object-usage for generating the object-usage scenario. It is more likely that the probability of an important object of an activity will be higher than that of an unimportant object.

## 8. Discussion

During configuration of the tool and testbed, an administrator should keep the followings in mind:

- One of the important issues of this tool is to provide the name of the activities, locations and objects because the efficiency of the activity generator depends on choosing the appropriate names. Wrong or malicious names of such would lead to error in generating a reliable dataset. Therefore, during the configuration of the tool, an administrator should choose short but appropriate names of activities, locations and objects.
- Choosing the right object is an important factor for reducing the noises of the dataset. For example, choosing the “shower faucet” in the bathroom would reduce noise of “Bathing”, because it is highly likely that “shower faucet” will be used while “Bathing”.
- The values for *n* and *T* should be chosen properly. It is quite natural that, we can increase the input flexibility in choosing lower value for *n*. However, such lower value of *n* may interfere with the projection of real object-usage scenario. Therefore, the administrator is advised to choose a value for *n* by considering a trade-off between the two performance factors.

Although the proposed tool can generate a reliable dataset with the help of participant’s input and the web activity data, it could not be considered as a replacement of a dataset acquired in a real testbed. A dataset acquired from a real testbed is always preferable not only for training an activity recognition system but also for evaluating its performance.

The activity data collection is passive, in a sense that the system hugely relies on the user for annotation as well as performing activity in a pre-defined sequence of object usage. But a user might end up giving incomplete, wrong or malicious object-usage scenario. Although the activity generator will try to remove the noises, it may end up generating inaccurate activity log.

The datasets provided by different people may suffer from inconsistency as different users execute and describe activity differently. However, as long as the activity descriptions are reliable, the datasets are helpful for training and evaluating a system’s performance. More and more of such datasets are indeed required to develop a robust and general-purpose activity recognition system.

## 9. Conclusions and Future Work

In this paper, we have proposed a web-based activity data collection tool which can be used as a rapid alternative of the state-of-the-art activity data collection. The tool provides a series of web-based interfaces through which an administrator (or a user with domain knowledge) can configure the tool as well as the testbed. It also provides a web-based EST by which a user can provide his/her activity experience. For providing an experience the user simply needs to provide the duration of an activity along with partial timings (or noisy timings) of the set of object-usage. The tool has an intelligent activity generator which generates the actual activity log by reducing the noises of user’s input. The activity generator uses the web activity data to generate such activity log. The web activity data is mined by an activity mining engine. We have shown that it is possible to accumulate diverse set of real-world activity data using the proposed tool. We have performed two experiments to validate the tool’s performance in producing reliable datasets.

Even though the activity generation component could provide close to real-world object-usage scenario, it may fail to furnish the exact object-usage sequence in parallel environment (when two or more objects are used within same time duration). The future version of this component would overcome such a drawback. We are planning to use object-usage sequence information mined from the web, using an improved mining algorithm.

## Figures and Tables

**Table 1.** The EST representation of participant’s input regarding vacuuming.

	1st slot (*d*_1_)	2nd slot (*d*_2_)	3rd slot (*d*_3_)	4th slot (*d*_4_)	5th slot (*d*_5_)
Closet					
Vacuum					
Burner					

**Table 2.** The IAGC representation of participant’s input for vacuuming.

	1st slot (*d*_1_)				2nd slot (*d*_2_)				3rd slot (*d*_3_)				4th slot (*d*_4_)				5th slot (*d*_5_)			
	*t*_1_	*t*_2_	*t*_3_	*t*_4_	*t*_1_	*t*_2_	*t*_3_	*t*_4_	*t*_1_	*t*_2_	*t*_3_	*t*_4_	*t*_1_	*t*_2_	*t*_3_	*t*_4_	*t*_1_	*t*_2_	*t*_3_	*t*_4_
Closet																				
Vacuum																				
Burner																				

**Table 3.** List of objects to acquire object-usage information.

Location name	Number of objects	Object name
Bedroom	4	Cabinet, Door, Light switch, Mattress
Hallway	2	Closet, Door
Kitchen	14	Burner, Cabinet, Coffee machine, Stove, Exhaust Fan, Freezer, Laundry Dryer, Oven, Microwave, Refrigerator, Faucet, Sink Faucet, Toaster, Washing Machine
Living room	3	Door, Television, Light switch
Bathroom	7	Bathtub, Door, Exhaust Fan, Light switch, Faucet, Sink Faucet, Toilet Flush

**Table 4.** An example dataset.

Activity table		
Start time	End time	ID
01-April-2010 22:30:00	02-April-2010 06:31:00	45
02-April-2010 06:35:00	02-April-2010 06:42:00	39
02-April-2010 06:43:00	02-April-2010 06:58:00	40
02-April-2010 07:02:00	02-April-2010 07:17:00	48

Object-usage table		
Start time	End time	ID
01-April-2010 22:30:00	01-April-2010 22:31:00	10
01-April-2010 22:31:00	01-April-2010 22:32:00	10
01-April-2010 22:32:00	01-April-2010 22:33:00	19
01-April-2010 22:33:00	01-April-2010 22:34:00	10

**Table 5.** The confusion matrix produced by the classifier when it is trained using the web activity data.

	Going out	Toileting	Bathing	Dressing	Sleeping	Breakfast	Dinner	Doing laundry	Watching TV
Going out	1.00	0.00	0.00	0.00	0.00	0.00	0.00	0.00	0.00
Toileting	0.04	0.30	0.61	0.00	0.04	0.00	0.00	0.00	0.00
Bathing	0.00	0.20	0.80	0.00	0.00	0.00	0.00	0.00	0.00
Dressing	0.40	0.00	0.05	0.55	0.00	0.00	0.00	0.00	0.00
Sleeping	0.00	0.08	0.00	0.00	0.92	0.00	0.00	0.00	0.00
Breakfast	0.00	0.00	0.00	0.00	0.06	0.94	0.00	0.00	0.00
Dinner	0.00	0.00	0.19	0.05	0.00	0.10	0.67	0.00	0.00
Doing laundry	0.00	0.00	0.00	0.00	0.00	0.10	0.00	0.90	0.00
Watching TV	0.00	0.00	0.00	0.00	0.00	0.00	0.00	0.00	1.00

**Table 6.** Real-life database generation accuracy.

	Going out	Toileting	Bathing	Sleeping	Breakfast	Dinner	Drink
n	2	5	5	2	5	5	2
Accuracy	0.72	0.97	0.88	0.61	0.94	0.69	0.96
